# Supplementary material for: Learning to predict synchronization of coupled oscillators on randomly generated graphs
Source: Sci Rep. 2022 Sep 5;12:15056. doi: 10.1038/s41598-022-18953-8 (PMC9445105; doi:10.1038/s41598-022-18953-8)
Supplement: Supplementary file 1 — Supplementary Information. [file 41598_2022_18953_MOESM1_ESM.pdf]

Additional information

Three models for coupled oscillators

The *Kuramoto model* (KM) is one of the most well-studied models for coupled oscillators with continuous states[1–4]. Namely, fix a graph $G=(V,E)$ and the continuous state space $\Omega=\mathbb{R}/2\pi\mathbb{Z}$. A given initial phase configuration $X_0 : V \to \Omega$ evolves via the following systems of ordinary differential equations

$$\frac{d}{dt}X_t(v) = \omega_v + \sum_{u\in N(v)} K\sin(X_t(u)-X_t(v)), \tag{1}$$

for all nodes $v \in V$, where $N(v)$ denotes the set of neighbors of v in G, K denotes the *coupling strength*, and ω_v denotes the *intrinsic frequency* of v. Since we are interested in the dichotomy between synchronization and non-synchronization, we will be assuming identical intrinsic frequencies, which can be assumed to be zero without loss of generality by using a rotating frame. Note that synchronization is an *absorbing state* under this assumption, that is, X_t is synchronized for all $t \geq s$ if X_s is synchronized. In order to simulate KM, we use the following discretization

$$X_{t+h}(v) - X_t(v) = h\left(\sum_{u\in N(v)} K\sin(X_t(u)-X_t(v))\right), \tag{2}$$

with step size $h=0.05$. Accordingly, an 'iteration' for KM is a single application of the difference equation (2). After a change of time scale, we write X_k for the configuration obtained after k iterations.

We also consider two discrete models for coupled oscillators. Let $\Omega=\mathbb{N}/\kappa\mathbb{Z}$ be the κ-state color wheel for some integer $\kappa \geq 3$. The κ-color *Firefly Cellular Automata* (FCA) is a discrete-state discrete-time model for inhibitory Pulse Coupled Oscillators (PCOs) introduced by Lyu[5]. The intuition is that we view each node as an identical oscillator (firefly) of κ-states, which *blinks* whenever it returns to a designated *blinking color* $b(\kappa)=\lfloor\frac{\kappa-1}{2}\rfloor$; nodes with post-blinking color $c \in (b(\kappa),\kappa-1]$ in contact with at least one blinking neighbor do not advance, as if their phase update is being inhibited by the blinking neighbors, and otherwise advance to the next color (see Figure 1). More precisely, the coupling for FCA is defined as the following equation

$$X_{t+1}(v) = \begin{cases} X_t(v) & \text{if } X_t(v) > b(\kappa) \text{ and } X_t(u) = b(\kappa) \text{ for some } u \in N(v) \\ X_t(v)+1 & \text{otherwise.} \end{cases} \tag{3}$$

For visualization purposes, it is convenient to consider the equivalent dynamics of 'centered colorings' $\bar{X}_t := X_t - t \pmod{\kappa}$, so that if X_t synchronizes, then $\bar{X}_t$ converges to a constant function. In fact, FCA dynamics are displayed in this way in Figures 1 and 2 in the main text.

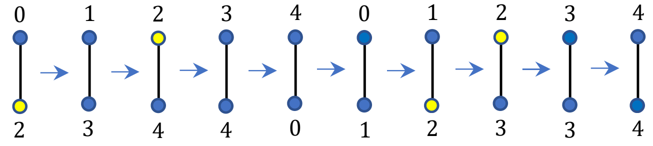

Figure S1. An example of 5-color FCA dynamics on two connected nodes. $b(5)=2$ is the blinking color shown in yellow.

On the other hand, the *Greenberg-Hastings model* (GHM) is a discrete model for neural networks[6] introduced in 1987 by Greenberg and Hastings, where nodes of color 0 and 1 are called 'rested' and 'excited', respectively, and of any other color called 'refractory'. As in biological neural networks, rested neurons gets excited by neighboring excited nodes, and once excited, it has to spend some time in rested states to come back to the rested state again. More precisely, the coupling for GHM is defined as

$$X_{t+1}(v) = \begin{cases} 0 & \text{if } X_t(v)=0 \text{ and } X_t(u)\neq 1 \text{ for all } u\in N(v) \\ 1 & \text{if } X_t(v)=0 \text{ and } X_t(u)=1 \text{ for some } u\in N(v) \\ X_t(v)+1 & \text{otherwise.} \end{cases} \tag{4}$$

For GHM, note that X_s is synchronized if and only if $X_t \equiv 0$ for all $t \geq s+\kappa$.

In all experiments in this paper, we consider $\kappa=5$ instances of FCA and GHM. From here and hereafter, by FCA and GHM we mean the 5-color FCA and 5-color GHM, respectively.

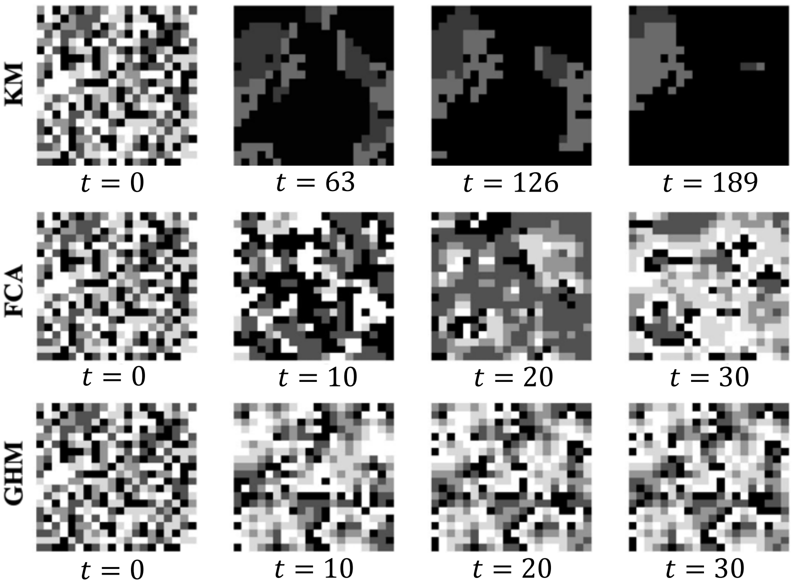

Figure S2. Simulation of KM, FCA, and GHM on the same underlying graph and initial configuration. The graph is generated by adding 80 edges uniformly at random into 20×20 square lattice. Each square heap map represents a phase configuration X_t at the corresponding iteration t shown below.

While all three models tend to synchronize locally their global behavior on the same graph and initial configuration evolve quite differently, as seen in Figure 2. There, we simulate each system on the same graph, a 20x20 lattice with an additional 80 edges added uniformly at random. A single initial phase concentration X_0 is chosen by assigning each node with a uniformly randomly chosen state from $\{0,1,2,3,4\}$. This initial configuration is evolved through three different models: FCA, GHM and KM.

This suggests that it is not feasible to predict synchronization for all three dynamics in the same way. Furthermore, the inclusion of random edges to the square lattice may disrupt some well-known behavior (e.g., spiral waves for coupled oscillators in 2D lattice[7]) and result in unpredictable dynamics, analytically so. Hence, predicting synchronization on fully heterogeneous sets of graphs is a difficult task since keeping track of local interactions of oscillators is challenging to do when the overall structures of graphs within the heterogeneous set correspondingly have highly irregular and diverse couplings.

A simple lemma about Greenberg-Hastings model on a ring

Lemma. *Consider the κ-color Greenberg-Hastings model* (4) *on a path of n nodes. Then for arbitrary initial coloring, $\kappa \geq 3$, and $n \geq 1$, the system will always converge to an all-zero configuration in $n+\kappa$ iterations.*

Proof. We use a back-tracking argument that was used to analyze FCA in[5,8,9]. Order the nodes in the path as $1,\dots,n$ from left to right so that nodes that differ by 1 are adjacent. Let $X_t : \{1\dots,n\} \to \{0,1,\dots,\kappa\}$ denote the color configuration at time t. Fix $T > \kappa$ and suppose that X_T is not identically zero. Then necessarily $X_{T-\kappa}$ should have at least one 1.

Suppose that node i has color 1 at time $T-\kappa$. Then by the dynamics, one of its two neighbors ($i-1$ or $i+1$) should have color 1 at time $T-\kappa-1$. Suppose that node $i+1$ has color 1 at time $T-\kappa-1$. By a similar argument, one of its two neighbors, now i or $i+2$, should have color 1 at time $T-\kappa-2$. But since $X_{T-\kappa-1}(i)=1$, we should have $X_{T-\kappa-2}(i)=0$, so we must have $X_{T-\kappa-1}(i+2)=1$. Repeating this argument, we see that backtracking in time, the color 1 at node 0 at time $T-\kappa$ should travel at constant unit velocity, that is, $X_{T-\kappa-s}(i+1+s)=1$ for all s such that $i+1+s \leq n$. It follows that $X_{T-\kappa-(n-i-1)}(n-1)=0$ and $X_{T-\kappa-(n-i-1)}(n)=1$. But this yields a contradiction since then at time $T-\kappa-(n-i)$, node n has color 0 and its only neighbor $n-1$ cannot have color 1 to excite node n. By a similar argument, if node $i-1$ has color 1 at time $T-\kappa-1$, then the dynamics can only be back-tracked through time $T-\kappa-i+1$. This shows that $X_{T-\kappa}$ cannot have color 1 if $T-\kappa > n$. We conclude that that X_T should be identically zero of $T > \kappa+n$. □.

Machine learning algorithms for binary classification

Different machine learning algorithms were employed to solve the problem of predicting whether or not a given graph and initial coloring for a system of coupled oscillators will tend to a synchronizing or non-synchronizing trajectory.

Random Forest (RF)[10] A random forest (RF) is a form of ensemble learning that produces decision trees, and in which these trees themselves are generated using random feature vectors that are sampled independently from a distribution.

Our implementation imposes a limit on the maximum amount of features used per tree to be the square root of the total amount of features in our dataset, $\sqrt{p}$ (where p is the number of features), and uses 100 estimator trees with iterates terminating at complete node purity.

Gradient Boosting (GB)[11] Gradient boosting (GB) is an ensemble learning algorithm for classification tasks similar to RFs where a loss function is minimized by a collection of decision trees to form a strong learner from a group of weak learners. The main difference is that the trees are not trained simultaneously, as in RFs, but added iteratively, so that the current loss is reduced cumulatively so in each iteration for each tree.

Our implementation uses a learning rate of 0.4, 100 estimator trees, and the square root of total number of features per tree when searching for splits.

Feed-forward Neural Networks (FFNN)[12] Feedforward Neural Networks (FFNN) form a class of function approximators consisting of multiple layers of connected linear and non-linear activation functions[13,14]. The linear maps are parameterized by 'weights', which are subject to training on a given dataset. The algorithm works by iterating two phases. The first phase is the 'feed-forward' phase; the input is mapped to an output by applying the layers of linear and non-linear activation functions with the current weights. The loss between the output and the target labels are then computed. The second phase is the 'back-propagation' phase; the weights are modified in the direction that reduces the computed loss from the forward propagation phase.

Our implementation of an FFNN uses a learning rate of 0.01, cross-entropy loss, 4 fully connected linear layers, a hidden layer size of 100, ReLU activation, batch normalization, batch size 256, and dropout of 0.25 probability across 35 epochs. See[15] for backgrounds and jargons.

GraphLRCN A Long-term Recurrent Convolutional Network (LRCN)[16] is a neural network architecture that has been developed for video data classification by combining convolutional neural networks[17] and a special type of recurrent neural network known as the Long Short-Term Memory network (LSTM-Net)[18]. We propose a variant of LRCN that is suitable for learning to predict dynamics on graphs, which we call the *GraphLRCN*. The idea is to encode each configuration X_t on a graph G as a weighted adjacency matrix $\Delta(X_t)$ of G. Then the dynamics $(X_t)_{0\le t\le r}$ can be turned into a sequence of square matrices, which can be viewed as video data subject to classification.

Here we give a precise definition of the encoding $X_t \mapsto \Delta(X_t)$. Given a κ-coloring X on a graph $G=(V,E)$, we define the associated *color displacement matrix* $\Delta = \Delta(X) \in (\mathbb{Z}_\kappa)^{|V|\times|V|}$ as

$$\Delta(i,j) = \min\begin{pmatrix} X(i)-X(j) \ (\text{mod } \kappa), \\ X(j)-X(i)(\text{mod } \kappa) \end{pmatrix} \mathbf{1}((i,j)\in E). \tag{5}$$

If $X : V \to [0,2\pi)$ is a configuration for KM, we define the associated color displacement matrix $\Delta(X)$ similarly as above by replacing mod κ with mod 2π. One can think of $\Delta(X)$ as the adjacency matrix of G weighted by the color differences assigned by X on the edges. An important feature of this encoding is that $\Delta(X)(i,j)$ is nonzero if and only if nodes i,j are adjacent in G *and* have different colors $X(i)\neq X(j)$. For instance, if X is synchronized then $\Delta(X)$ is the zero matrix. Additionally, since the convolutional block components learn physical location-based associations in the matrix, we applied the reverse Cuthill-Mckee algorithm[19] on the sequences of adjacency matrices to reduce our matrix bandwidths and augment these physical associations in our representations. In combination, the techniques mentioned are intended to reduce the amount of unnecessary information and noise in the encoding for the purpose of learning to predict synchronization. Our implementation uses a learning rate of 0.01, a batch size of 2^{11}, and batch normalization across 25 epochs.

Additional figures

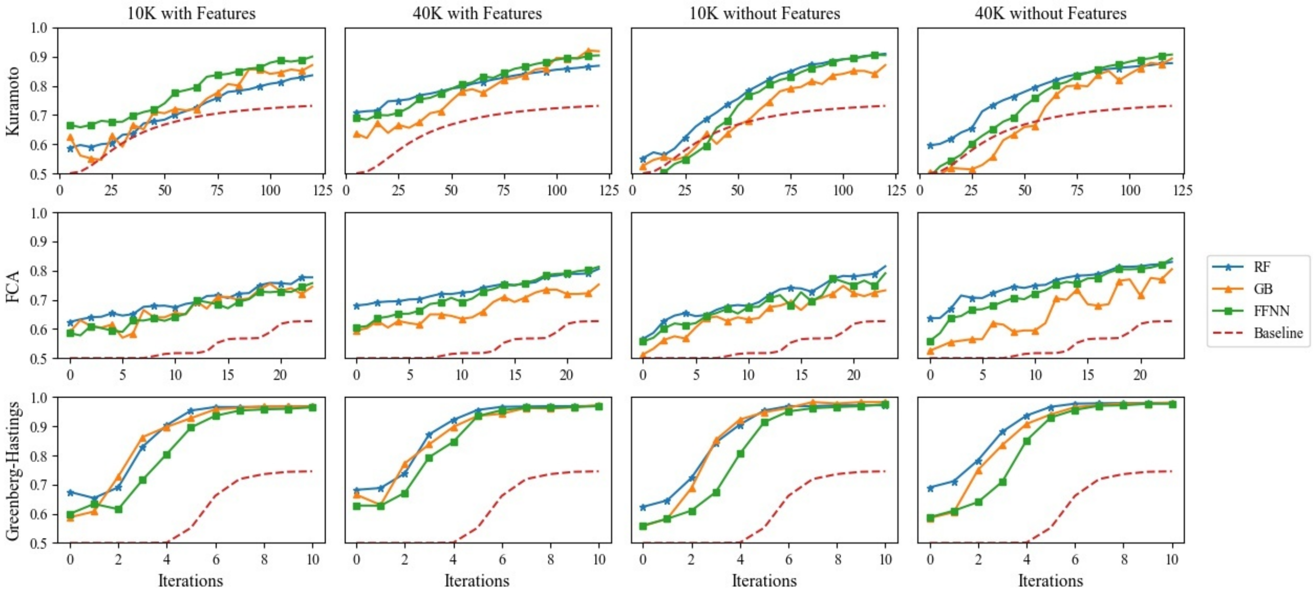

Figure S3. Supplementary experiment to Figure 5 in the main text to show sensitivity of our synchronization prediction methods to the size of training dataset. All settings are identical to the one in Figure 3 in the main text, except here we use smaller subsampled training set of sizes 10K and 40K for each classes of synchronizing and non-synchronizing examples, averaged over five runs. The first two columns use datasets with 15 node graphs, while the last two columns use datasets with 30 node graphs. Note that the full dataset used in Figure 3 in the main text uses 80K examples. Even trained with these smaller training set, we observe that most of our prediction algorithms significantly outperforms the baseline, although the performance does decrease in comparison to the main experiment in Figure 5 in the main text. (Note: This was averaged across 5 runs to address the randomness associated with subsampling).

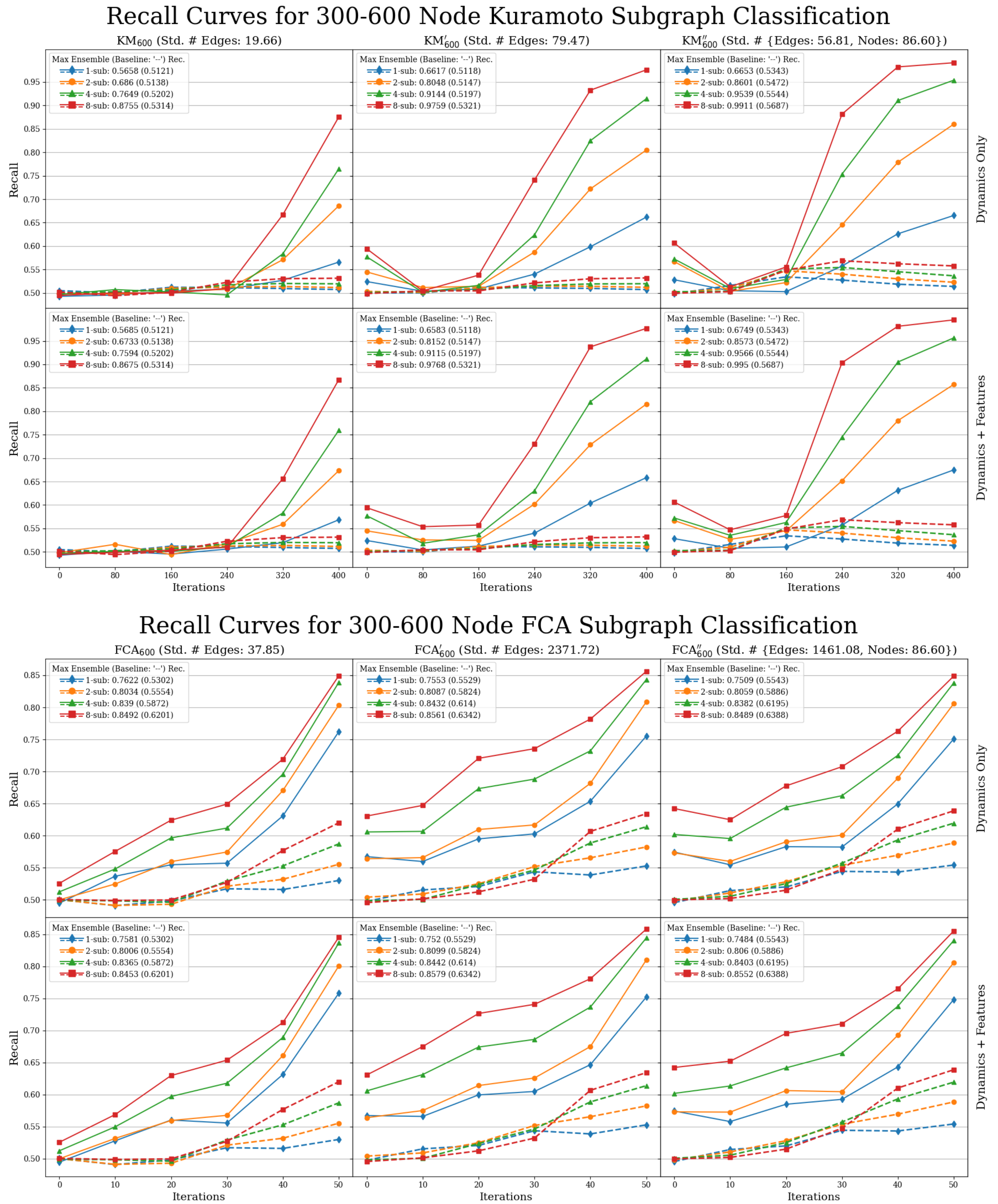

Figure S4. Recall curves for predicting synchronization of both Kuramoto (top) and 5-color FCA (bottom) on 600-node graphs from dynamics observed from $k \in \{1,2,4,8\}$ subgraphs of 30 nodes. All plots observe the performance of both the ensemble machine learning (solid) and baseline (dashed) recall scores over increasing amounts of training iterations $r \in \{0,80,240,320,400\}$. The first row shows results using only dynamics whereas the second row includes both the dynamics and graph features. Maximum recall scores for using k subgraphs are given by 'k-sub: Acc. (Baseline Acc.)'

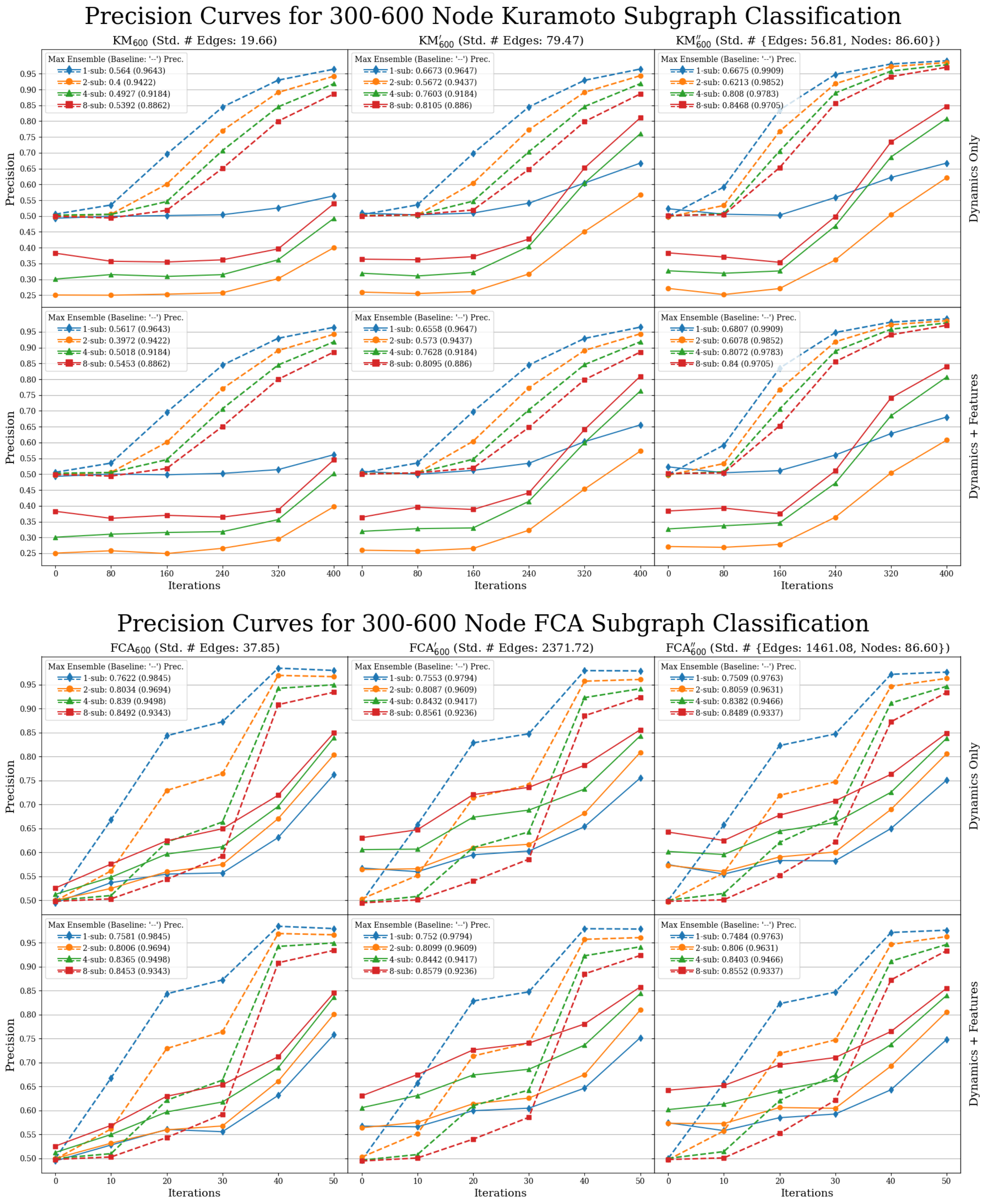

Figure S5. Precision curves for predicting synchronization of both Kuramoto (top) and 5-color FCA (bottom) on 600-node graphs from dynamics observed from $k \in \{1,2,4,8\}$ subgraphs of 30 nodes. All plots observe the performance of both the ensemble machine learning (solid) and baseline (dashed) precision scores over increasing amounts of training iterations $r \in \{0,80,240,320,400\}$. The first row shows results using only dynamics whereas the second row includes both the dynamics and graph features. Maximum precision scores for using k subgraphs are given by 'k-sub: Acc. (Baseline Acc.)'

References

1. Kuramoto, Y. *Chemical oscillations, waves, and turbulence* (Courier Corporation, 2003).
2. Acebrón, J. A., Bonilla, L. L., Vicente, C. J. P., Ritort, F. & Spigler, R. The kuramoto model: A simple paradigm for synchronization phenomena. *Rev. modern physics* **77**, 137 (2005).
3. Strogatz, S. H. From kuramoto to crawford: exploring the onset of synchronization in populations of coupled oscillators. *Phys. D: Nonlinear Phenom.* **143**, 1–20 (2000).
4. Lee, T. E., Tam, H., Refael, G., Rogers, J. L. & Cross, M. Vortices and the entrainment transition in the two-dimensional kuramoto model. *Phys. Rev. E* **82**, 036202 (2010).
5. Lyu, H. Synchronization of finite-state pulse-coupled oscillators. *Phys. D: Nonlinear Phenom.* **303**, 28–38 (2015).
6. Greenberg, J. M. & Hastings, S. P. Spatial patterns for discrete models of diffusion in excitable media. *SIAM J. on Appl. Math.* **34**, 515–523 (1978).
7. Martens, E. A., Laing, C. R. & Strogatz, S. H. Solvable model of spiral wave chimeras. *Phys. review letters* **104**, 044101 (2010).
8. Lyu, H. Phase transition in firefly cellular automata on finite trees. *arXiv preprint arXiv:1610.00837* (2016).
9. Lyu, H. & Sivakoff, D. Persistence of sums of correlated increments and clustering in cellular automata. *Stoch. Process. their Appl.* **129**, 1132–1152 (2019).
10. Breiman, L. Random forests. *Mach. learning* **45**, 5–32 (2001).
11. Friedman, J. H. Stochastic gradient boosting. *Comput. statistics & data analysis* **38**, 367–378 (2002).
12. Bishop, C. M. & Nasrabadi, N. M. *Pattern recognition and machine learning*, vol. 4 (Springer, 2006).
13. Csáji, B. C. *et al.* Approximation with artificial neural networks. *Fac. Sci. Etvs Lornd Univ. Hungary* **24**, 7 (2001).
14. Hornik, K. Approximation capabilities of multilayer feedforward networks. *Neural networks* **4**, 251–257 (1991).
15. Svozil, D., Kvasnicka, V. & Pospichal, J. Introduction to multi-layer feed-forward neural networks. *Chemom. intelligent laboratory systems* **39**, 43–62 (1997).
16. Donahue, J. *et al.* Long-term recurrent convolutional networks for visual recognition and description. In *Proceedings of the IEEE conference on computer vision and pattern recognition*, 2625–2634 (2015).
17. Krizhevsky, A., Sutskever, I. & Hinton, G. E. Imagenet classification with deep convolutional neural networks. *Adv. neural information processing systems* **25** (2012).
18. Hochreiter, S. & Schmidhuber, J. Long short-term memory. *Neural computation* **9**, 1735–1780 (1997).
19. Cuthill, E. & McKee, J. Reducing the bandwidth of sparse symmetric matrices. In *Proceedings of the 1969 24th national conference*, 157–172 (1969).
